# Altered Levels of Mitochondrial DNA Are Associated with Female Age, Aneuploidy, and Provide an Independent Measure of Embryonic Implantation Potential

**DOI:** 10.1371/journal.pgen.1005241

**Published:** 2015-06-03

**Authors:** Elpida Fragouli, Katharina Spath, Samer Alfarawati, Fiona Kaper, Andrew Craig, Claude-Edouard Michel, Felix Kokocinski, Jacques Cohen, Santiago Munne, Dagan Wells

**Affiliations:** 1 Reprogenetics UK, Oxford, United Kingdom; 2 Nuffield Department of Obstetrics and Gynaecology, University of Oxford, Oxford, United Kingdom; 3 Illumina, San Diego, California, United States of America; 4 Illumina, Cambridge, United Kingdom; 5 Reprogenetics LLC, Livingston, New Jersey, United States of America; Stanford University Medical Center, UNITED STATES

## Abstract

Mitochondria play a vital role in embryo development. They are the principal site of energy production and have various other critical cellular functions. Despite the importance of this organelle, little is known about the extent of variation in mitochondrial DNA (mtDNA) between individual human embryos prior to implantation. This study investigated the biological and clinical relevance of the quantity of mtDNA in 379 embryos. These were examined via a combination of microarray comparative genomic hybridisation (aCGH), quantitative PCR and next generation sequencing (NGS), providing information on chromosomal status, amount of mtDNA, and presence of mutations in the mitochondrial genome. The quantity of mtDNA was significantly higher in embryos from older women (P=0.003). Additionally, mtDNA levels were elevated in aneuploid embryos, independent of age (P=0.025). Assessment of clinical outcomes after transfer of euploid embryos to the uterus revealed that blastocysts that successfully implanted tended to contain lower mtDNA quantities than those failing to implant (P=0.007). Importantly, an mtDNA quantity threshold was established, above which implantation was never observed. Subsequently, the predictive value of this threshold was confirmed in an independent blinded prospective study, indicating that abnormal mtDNA levels are present in 30% of non-implanting euploid embryos, but are not seen in embryos forming a viable pregnancy. NGS did not reveal any increase in mutation in blastocysts with elevated mtDNA levels. The results of this study suggest that increased mtDNA may be related to elevated metabolism and are associated with reduced viability, a possibility consistent with the ‘quiet embryo’ hypothesis. Importantly, the findings suggest a potential role for mitochondria in female reproductive aging and the genesis of aneuploidy. Of clinical significance, we propose that mtDNA content represents a novel biomarker with potential value for in vitro fertilisation (IVF) treatment, revealing chromosomally normal blastocysts incapable of producing a viable pregnancy.

## Author Summary

Mitochondria are small membrane-enclosed structures and are found inside the cells of the body. Mitochondria actively participate in cellular life, and their main function is to generate energy which is used by the cell. For this reason mitochondria are considered as the powerhouses of cells. Unlike other cellular organelles, mitochondria contain their own DNA (mtDNA). MtDNA carries important genetic information concerning cellular metabolism and the generation of energy. It has been suggested that mitochondria and mtDNA could be of significance during early embryo development. Our work confirms this hypothesis. Specifically, our findings implicate mitochondria and their genome in female reproductive aging and the generation of embryonic chromosome abnormalities. Importantly, we describe a direct relationship between mtDNA quantity and the potential of an embryo to successfully become a baby. We propose that assessment of mtDNA quantity could be a novel way of identifying embryos with the highest ability to lead to healthy pregnancies and live births.

## Introduction

Mitochondria are involved in the regulation of multiple essential cellular processes, such as apoptosis, amino acid synthesis, calcium homeostasis, and the generation of energy in the form of ATP via the process of oxidative phosphorylation (OXPHOS) [1–5]. For this reason mitochondria are considered as the principal cellular power houses. They are unique compared to other organelles in animal cells in that they contain one or more copies of their own genome. The mitochondrial DNA (mtDNA) is circular and composed of 16.6 kb of double stranded DNA. Genes encoded by this DNA molecule have direct roles in cellular metabolism, producing subunits of several complexes with key roles within the electron transport chain (ETC) [6]. Complexes encoded by the mitochondrial genome, along with other ETC components, are situated in the inner mitochondrial membrane and are vital for the production of ATP in the cell. Additionally, mtDNA encodes some of the components of the organelle’s transcriptional and translational machinery including 22 tRNAs and 2 rRNAs, with the remainder being encoded by the nuclear genome [6]. It has been shown that cells are capable of redistributing their mitochondria so as to replace damaged organelles, and adjust to variation in intracellular energy requirements [7].

The mitochondrial content of mammalian cells ranges from a few hundred to thousands, determined by the cell’s volume and energy needs. The human mature oocyte is among the cell types with the highest content for both mitochondria and mtDNA [1]. Oocyte mitochondrial replication begins during fetal development with cells of the oogonia containing approximately 200 mitochondria [reviewed in 8]. Replication continues in synchrony with maturation, so that just before fertilisation an oocyte arrested at metaphase II contains approximately 100,000 mitochondria and between 50,000 and 550,000 copies of the mtDNA [1, 9–13].

Mammalian embryos inherit mitochondria (and thus mtDNA) exclusively from the population found in the oocyte just prior to fertilisation. Data from quantification of mtDNA in human cleavage stage embryos suggests that amounts remain stable during the first three days of preimplantation development [1, 12–16]. Significant replication of mtDNA is not thought to be initiated until after the embryo has undergone the first cellular differentiation into trophectoderm (TE) and inner cell mass and has become a blastocyst [3, 8].

Preimplantation development is a dynamic and energy demanding process. Early embryos require adequate energy levels so that they can successfully progress through each cell division. Existing data suggest that correct oocyte mitochondrial function and mtDNA gene expression are essential during these early stages of life. Specifically, an association has been shown between the ATP content of human oocytes, the developmental potential of an embryo, and the outcome of an IVF cycle [17].

Since mitochondrial functions are critical during the first few days of life, we were interested in carrying out a thorough investigation of mtDNA in human preimplantation embryos which had successfully reached the blastocyst stage of development. Specifically, we examined the relationship between human blastocyst mtDNA content, female patient age, embryo chromosome status, viability and implantation potential. Additionally, we attempted to shed light on the stage of preimplantation development during which mtDNA replication is first up-regulated, with the potential to increase the mtDNA content of individual cells. As well as relative quantification of mtDNA, a detailed analysis of the mitochondrial genome was undertaken, searching for mutations, deletions and polymorphisms.

## Results

### Cytogenetic analysis of embryonic samples

A total of 39 cleavage stage embryos and 340 blastocysts, which had been cytogenetically tested, were studied during the course of this investigation. All of the cleavage stage embryos had been characterised as being chromosomally normal after microarray comparative genomic hybridisation (aCGH) analysis and transferred to the uterus. Of the blastocysts examined, 302 were analysed using aCGH, and 38 using next generation sequencing (NGS) methodology. Of these, 123 were determined to be aneuploid (99 via aCGH analysis and 24 via NGS analysis), while the remaining 217 were characterised as being chromosomally normal (203 via aCGH analysis and 14 via NGS analysis). One hundred and thirty one of the normal blastocysts and all 39 euploid cleavage stage embryos underwent uterine transfer. Embryo classification as chromosomally normal or aneuploid was based on results obtained after aCGH or NGS analysis of either a single blastomere (cleavage stage), or 5–10 TE cells (blastocysts).

### The effect of female age on mtDNA quantity

**Fig 1:**
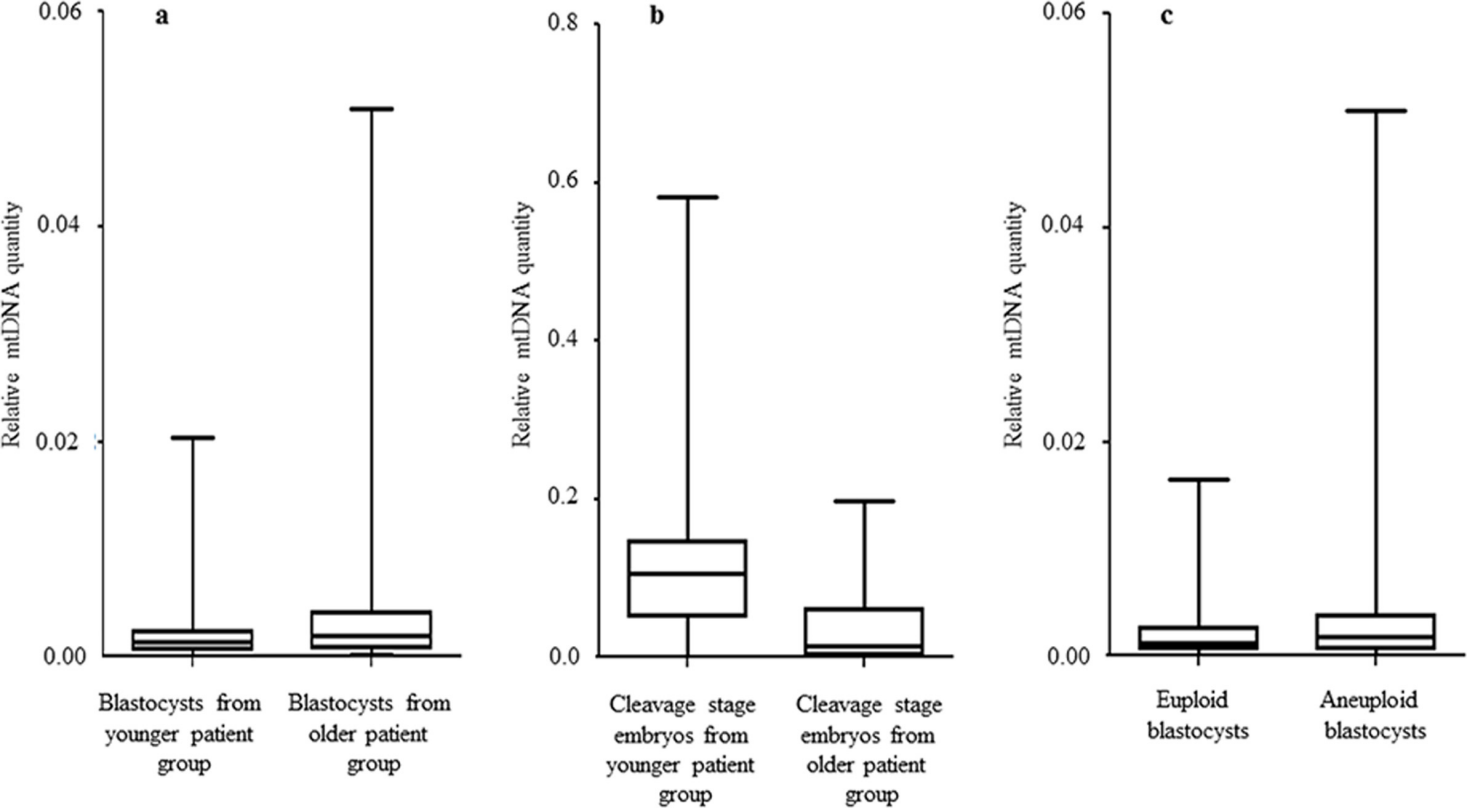
The relationship between mtDNA quantity, female age and embryo chromosome constitution. **a)** Data obtained during quantitative real-time PCR analysis of TE samples removed from 302 blastocysts demonstrated a statistically significant increase (P = 0.003) in the level of mtDNA in relation to advancing female age. This phenomenon was evident for both euploid and aneuploid blastocysts. **b)** Real-time PCR analysis of 39 blastomeres showed that cleavage stage embryos from reproductively younger women contained significantly (P = 0.01) higher mtDNA levels, compared to those generated by reproductively older women. **c)** Real-time PCR analysis of TE samples also demonstrated that aneuploid blastocysts (n = 99) contained significantly (P = 0.025) larger quantities of mtDNA at all ages, compared to those that were euploid (n = 203). Statistical analysis of mtDNA values took place with the use of unpaired two-tailed t-tests.

The relative amount of mtDNA was assessed in relation to female age. Specifically, an initial comparison of 148 blastocysts generated by a reproductively younger group of women (average age 34.8 years, range 26–37 years) and the154blastocysts generated by a reproductively older group (average age 39.8years, range 38–42 years) was undertaken with the use of real-time PCR. Data analysis clearly showed a statistically significant increase (P = 0.003) in the amount of mtDNA in blastocysts from the reproductively older women. This phenomenon was evident when all blastocysts were considered together, but was also apparent if chromosomally normal and abnormal embryos were considered separately (P = 0.018 and P = 0.05, respectively). The relative amounts of mtDNA in chromosomally normal and abnormal blastocysts for the female age groups under investigation are summarised in Table 1 and illustrated in Fig 1a.

**Table 1:** The average relative quantities of mtDNA observed in association to female age and blastocyst chromosome status.

Female age (years)	Number of embryos assessed	mtDNA quantity range/ euploid blastocysts	Average mtDNA quantity/ euploid blastocysts	mtDNA quantity range/ aneuploid blastocysts	Average mtDNA quantity/ aneuploid blastocysts
26	1 euploid	0.002273	N/A	N/A	N/A
29	1 euploid	0.0029	N/A	N/A	N/A
30	9 euploid 5 aneuploid	0.000065–0.0017	0.000482	0.000095–0.0069	0.00161
31	9 euploid 3 aneuploid	0.000093–0.00231	0.000998	0.00166–0.0044	0.00248
32	7 euploid 3 aneuploid	0.000318–0.00462	0.00137	0.000324–0.00275	0.00145
33	9 euploid 3 aneuploid	0.00013–0.002532	0.00115	0.00048–0.00218	0.00159
34	8 euploid 1 aneuploid	0.001–0.0034	0.0043	0.00244	0.00244
35	10 euploid 2 aneuploid	0.00076–0.00765	0.00265	0.00289–0.0034	0.00315
36	26 euploid 9 aneuploid	0.00032–0.00968	0.002	0.00026–0.0082	0.00212
37	29 euploid 12 aneuploid	0.000038–0.015	0.0023	0.000097–0.00416	0.00142
38	28 euploid 6 aneuploid	0.000294–0.01	0.00283	0.00037–0.007	0.00285
39	18 euploid 18 aneuploid	0.00063–0.0164	0.0025	0.00053–0.00722	0.00393
40	19 euploid 7 aneuploid	0.0004–0.0064	0.002	0.00036–0.0038	0.002
41	16 euploid 15 aneuploid	0.00025–0.006	0.0026	0.00027–0.05	0.0079
42	13 euploid 15 aneuploid	0.00013–0.012	0.0048	0.0001–0.016	0.0053

The mtDNA values (2-^Delta Delta Ct^) were obtained during real-time PCR analysis.

A significant difference (P = 0.01) in the levels of mtDNA according to female age was also observed at the cleavage stage. However, unlike the blastocyst stage, blastomeres removed from embryos generated by reproductively younger women (average age 33.7 years, range 29–37 years) were seen to contain higher mtDNA amounts, compared to those removed from embryos generated by reproductively older women (average age 39.2 years, range 38–42 years). These results are illustrated in Fig 1b and Table 2.

**Table 2:** The average relative quantities of mtDNA observed in association with female age at the cleavage stage.

Female age (years)	Number of embryos assessed	mtDNA quantity range	Average mtDNA quantity
29	1	0.1077	N/A
30	3	0.0673–0.5823	0.2912
33	4	0.1058–0.1716	0.139
34	3	0.00055–0.01389	0.0055
35	2	0.06480–0.1476	0.106
36	4	0.0504–0.1276	0.0831
37	2	0.00051–0.1176	0.059
38	9	0.00251–0.1967	0.0591
39	4	0.000297–0.00482	0.002
40	4	0.00927–0.0636	0.0254
42	3	0.00082–0.087	0.03

The mtDNA values (2-^Delta Delta Ct^) were obtained during real-time PCR analysis. All examined blastomeres were characterised as being chromosomally normal.

### The relationship between embryo chromosome constitution and mtDNA quantity

Chromosome abnormalities are extremely common during the earliest stages of embryo development, with rates decreasing post-implantation [18]. Real-time PCR assessment of mtDNA quantity in relation to chromosome status took place for a total of 203 normal and 99 aneuploid blastocyst stage embryos. TE samples from all these embryos were assessed with the use of aCGH. It was evident that chromosomally abnormal blastocysts tended to contain significantly larger amounts of mtDNA compared to those which were characterised as being euploid (P = 0.025) (Fig 1c).

**Fig 2:**
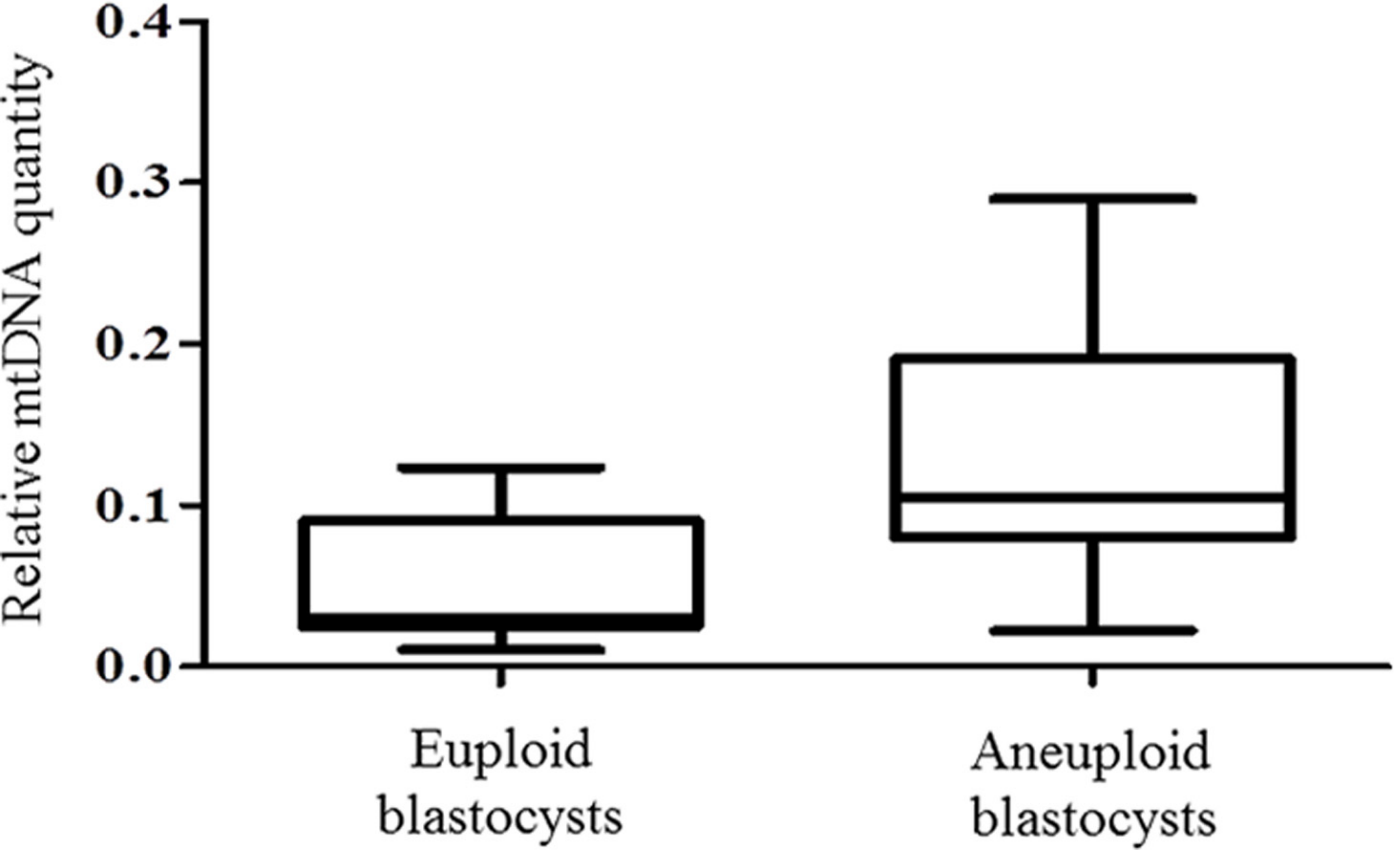
mtDNA quantification via NGS analysis of chromosomally normal and abnormal blastocysts. NGS analysis of TE samples biopsied from 38 embryos showed a statistically significant increase (P = 0.006) in the mtDNA levels occurring in the presence of chromosome errors.

To verify these results using an unrelated methodology, we applied a different type of whole genome amplification (WGA) method followed by NGS to TE biopsies derived from 38 additional blastocysts. The advantage of NGS technology is its capability to simultaneously examine nuclear and mitochondrial genomes. NGS analysis demonstrated that 14 of the blastocysts were euploid, whereas chromosome abnormalities were scored for the remaining 24. This finding was confirmed via aCGH conducted using separate aliquots of each WGA product. As with the real-time PCR results, statistical analysis of NGS data showed a significant increase (P = 0.006) in the quantity of mtDNA in aneuploid blastocysts compared to those that were chromosomally normal. This provided independent confirmation of the real-time PCR findings. The NGS mtDNA data are illustrated in Fig 2.

It should be noted that although mtDNA quantity increases with advancing female age, the relationship with aneuploidy appears to be an independent factor. Within any given age group mtDNA levels were higher, on average, for blastocysts that were chromosomally abnormal (Table 1).

### mtDNA copy number and the ability of blastocysts to establish a clinical pregnancy

In order to assess whether mtDNA content had an influence on the ability of an embryo to implant and initiate a pregnancy, we retrospectively analysed data obtained from single embryo transfers (SETs) with or without implantation, or double embryo transfers (DETs) which either led to dizygotic twins or no implantation. Specifically we examined the mtDNA content of 89 blastocysts, 81 of which were transferred in SETs with the remaining 8 being transferred in DETs. Eighty-five patients were included in this part of the study and the average female age was 38.3 years. Of the blastocysts transferred to these patients, 42 established an ongoing clinical pregnancy, while the remaining 47 failed to implant.

**Fig 3:**
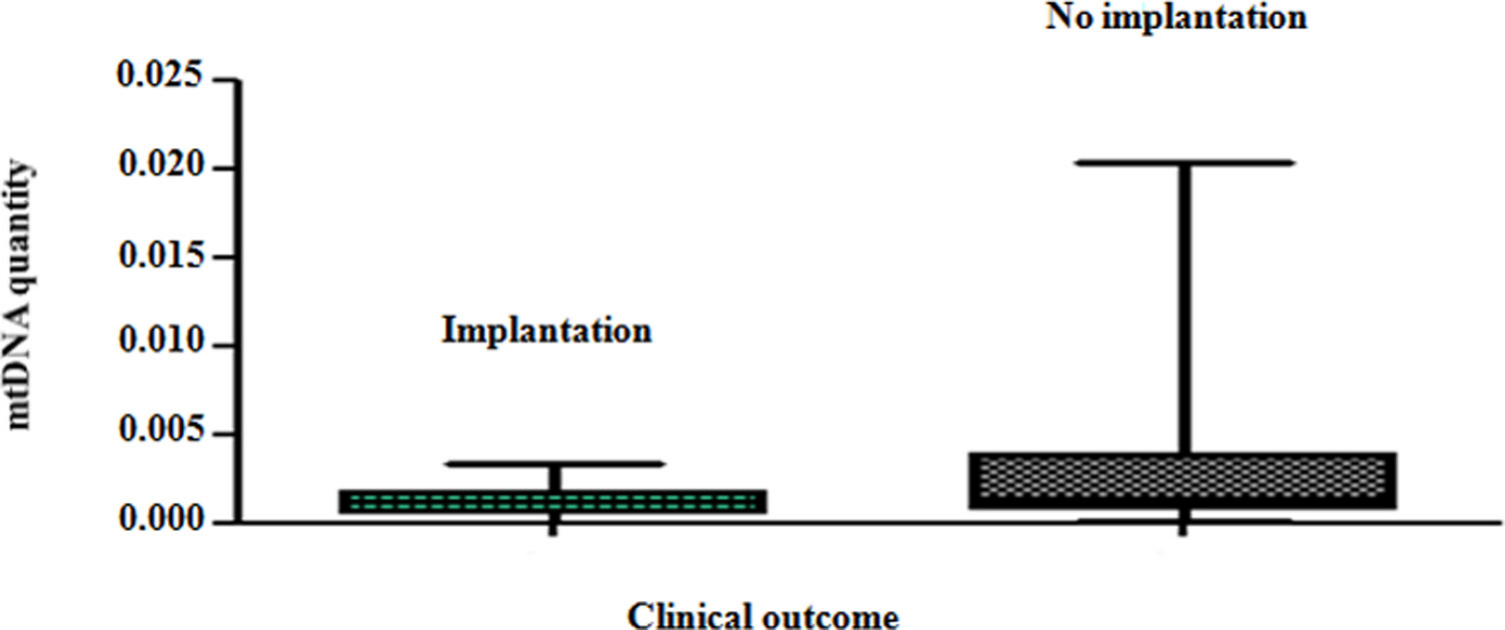
The mtDNA content of chromosomally normal blastocysts in relation to clinical outcome. On average, chromosomally normal blastocysts capable of establishing a clinical pregnancy contained significantly (P = 0.007) lower levels of mtDNA compared to chromosomally normal blastocysts that failed to do so.

Real-time PCR analysis clearly showed that blastocysts able to implant contained significantly lower amounts of mtDNA compared to those incapable of initiating a clinical pregnancy (P = 0.007). These results are summarised in Fig 3.

**Fig 4:**
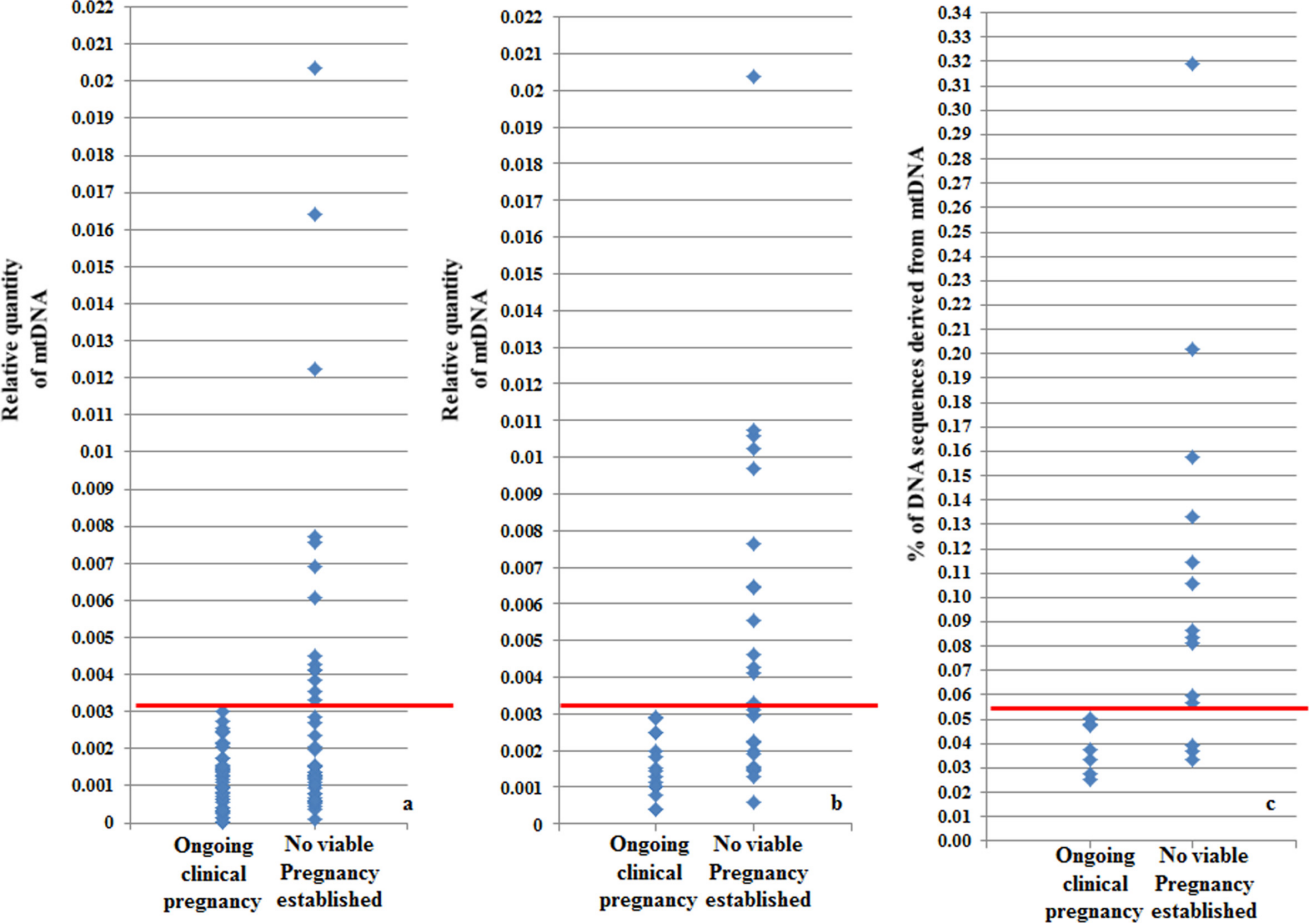
Blastocyst mtDNA quantity threshold in relation to clinical outcome. **a)** The mtDNA quantity viability threshold for euploid blastocysts, established via retrospective analysis of TE biopsies from transferred embryos with known outcomes. All blastocysts producing viable pregnancies contained mtDNA quantities below the 0.003 value (red line) whereas mtDNA quantities above this value were associated with failure to achieve an ongoing clinical pregnancy. **b)** Results of the prospective blinded study. The mtDNA threshold used was the same as that established in the retrospective study (4a). Validity was confirmed, since all blastocysts producing viable pregnancies contained mtDNA quantities below the cut-off (red line) and no blastocysts with mtDNA quantities above this value achieved an ongoing clinical pregnancy. **c)** NGS analysis of the mtDNA level in 23 euploid TE samples. The corresponding embryos were transferred during SET cycles, and clinical outcomes were known for 21 of them. As with the real-time PCR experiments, mtDNA levels were lower in the seven implanting embryos (note- the y-axis scale is different for NGS analyses and consequently cut-off values differ).

Analysis of the real-time PCR data obtained from implanting and non-implanting blastocysts allowed the establishment of an mtDNA quantity threshold above which implantation was never seen to occur. Specifically, 42/42 (100%) blastocysts which led to a clinical pregnancy contained relative mtDNA quantities lower than 0.003. Additionally, 14/14 (100%) of embryos with mtDNA quantities higher than 0.003 were unable to implant. These represented 30% (14 of 47) of the non-implanting blastocysts, while the remaining 70% (33 of 47) contained amounts of mtDNA below the threshold (Fig 4a). It is of note that the identified mtDNA quantity implantation threshold of 0.003 was independent of blastocyst morphology, age and the IVF clinic that produced the embryos.

To further evaluate the association between elevated mtDNA levels and implantation failure, we analysed 23 TE samples with the use of NGS. All of the embryos were euploid, and had previously been analysed via real-time PCR. The clinical outcome after transfer was known for 21 of the corresponding blastocysts. Seven of these led to pregnancies whereas the remaining 14 failed to implant. Of the 14 embryos which had not implanted, real-time PCR identified 9 containing mtDNA amounts higher than 0.003. NGS analysis confirmed the real-time PCR findings, clearly demonstrating increased quantities of mtDNA in non-implanting embryos compared to those shown to be viable. Elevated mtDNA quantities were also observed for an additional 3 of the TE samples for which clinical outcome was not known. These results are illustrated in Fig 4c and Table 3-

**Table 3:** mtDNA quantities and clinical outcomes of 23 TE samples assessed via real-time PCR and NGS.

TE sample	mtDNA quantity/ Real-time PCR (2-^Delta Delta Ct^)	MtDNA quantity/ Low (normal)/ High (abnormal)*	mtDNA quantity/ NGS (% of total reads)	MtDNA quantity/ Low (normal)/ High (abnormal)*	Clinical outcome after ET
A1	0.0004047	Low/ Normal	0.05	Low/ Normal	Implantation
A2	0.000131	Low/ Normal	0.03	Low/ Normal	Implantation
A3	0.001257	Low/ Normal	0.05	Low/ Normal	Implantation
A4	0.00103	Low/ Normal	0.04	Low/ Normal	No Implantation
A5	0.00026	Low/ Normal	0.03	Low/ Normal	Implantation
A6	0.000406	Low/ Normal	0.03	Low/ Normal	Implantation
A7	0.000669	Low/ Normal	0.03	Low/ Normal	No Implantation
A8	0.00151	Low/ Normal	0.04	Low/ Normal	Implantation
A9	0.00217	Low/ Normal	0.05	Low/ Normal	Implantation
A10	0.001208	Low/ Normal	0.06	Low/ Normal	No Implantation
A11	0.000548	Low/ Normal	0.04	Low/ Normal	No Implantation
A12	0.001932	Low/ Normal	0.04	Low/ Normal	Not known
B1	0.00341	High/ abnormal	0.08	High/ abnormal	No Implantation
B2	0.0164158	High/ abnormal	0.32	High/ abnormal	No Implantation
B3	0.00643	High/ abnormal	0.20	High/ abnormal	No Implantation
B4	0.000602	Low/ Normal	0.04	Low/ Normal	Not known
B5	0.01075	High/ abnormal	0.11	High/ abnormal	No Implantation
B6	0.00222	Low/ Normal	0.06	Low/ Normal	No Implantation
B7	0.0033	High/ abnormal	0.11	High/ abnormal	No Implantation
B8	0.00426	High/ abnormal	0.13	High/ abnormal	No Implantation
B9	0.00412	High/ abnormal	0.16	High/ abnormal	No Implantation
B10	0.0060	High/ abnormal	0.09	High/ abnormal	No Implantation
B11	0.0069	High/ abnormal	0.08	High/ abnormal	No Implantation

*The threshold for considering a sample to have elevated mtDNA levels, incompatible with implantation, was 0.003 for real-time PCR and 0.07 for NGS.

### Blinded prospective prediction of IVF outcome based upon mtDNA quantification

Following establishment of a viability threshold for mtDNA levels in blastocysts, based upon retrospective data analysis, we carried out a blinded prospective study to assess its predictive value. Quantification of mtDNA was carried out in TE biopsies from a total of 42 euploid blastocysts that had been selected for transfer to the uterus after chromosomal (aCGH) and morphological analyses. The average age of the women generating these embryos was 36.7 years (age range 26–42 years) and the couples were being treated in 6 different IVF clinics. Fifteen embryos were shown to have mtDNA levels above the 0.003 threshold and were therefore predicted to be associated with failure to establish a viable pregnancy (Fig 4b). Review of biochemical and ultrasound data a few weeks later, confirmed that none of these embryo transfers had resulted in a viable pregnancy. Thus the negative predictive value of the mtDNA analysis was 100%. The remaining 27 embryos had mtDNA quantities below 0.003 and were therefore predicted to have some potential for producing a child. After decoding of the blinded results, it was found that 16 of these embryos had ultimately established viable clinical pregnancies. Therefore, 59% of the embryos classified by mtDNA analysis as potentially viable created an ongoing clinical pregnancy. This contrasts to the 38% (16/42) pregnancy rate achieved for this cohort of embryos, transferred without reference to the mtDNA results. These results further confirmed our previous findings that embryos with high mtDNA quantities are incapable of forming a clinical pregnancy. Moreover, it was demonstrated that mtDNA quantification can be used as an effective biomarker to assist selection among euploid embryos.

### The origin of elevated levels of mtDNA in non-implanting embryos

In an attempt to shed light on whether the origin of excess mtDNA seen in non-implanting blastocysts was embryonic or was derived from the oocyte, we examined mtDNA quantities in blastomeres removed from 39 cleavage stage embryos. Mitochondrial DNA replication is not thought to occur until the blastocyst stage, so the levels of mtDNA detected at earlier developmental stages are expected to reflect those in the oocyte. All of the cleavage stage embryos considered in this part of the study had been characterised as euploid following blastomere aCGH analysis and had been transferred to the uterus. As far as the clinical outcome was concerned, 17 embryos were capable of implanting, leading to clinical pregnancies, while the remaining 22 did not implant.

It was evident that blastomeres contained much higher levels of mtDNA, compared to TE samples. This was not an unexpected finding, considering the much larger cytoplasmic volume of blastomeres in comparison to TE biopsies.

Assessment of the data obtained during this analysis showed that there was no significant difference in the quantities of mtDNA in blastomeres derived from embryos which implanted compared with those from embryos that failed to implant (P = 0.7). We therefore concluded that the increased mtDNA content seen in cells from a subset of non-viable blastocysts must originate after the cleavage stage. This conclusion is compatible with the notion that the first significant wave of mitochondrial genome replication begins after differentiation of embryonic cells into TE and inner cell mass is initiated.

### Mitochondrial genome analysis

One potential reason for altered mtDNA levels could be a proliferation of mitochondria as a compensatory response to the presence of defective organelles harbouring mutations in key genes. To explore this possibility NGS was used to sequence the entire mitochondrial genome of 23 TE samples. The samples were derived from chromosomally normal blastocysts, 9 of which had elevated quantities of mtDNA (initially determined using real-time PCR) and 14 that had mtDNA levels in the normal range (Table 3). The mitochondrial genome was sequenced to an average depth of ~150 reads, permitting mutation detection and an estimate of degree of heteroplasmy. Mutations, usually in heteroplasmic form, were seen to some extent in all samples, but were no more prevalent in blastocysts with high mtDNA levels than they were in embryos with lower quantities of mtDNA.

## Discussion

### Energy requirements of the developing preimplantation embryo

Early embryo development consists of a series of mitotic divisions and other cellular events requiring a supply of energy, principally in the form of ATP generated by mitochondria [19]. During the blastocyst stage, ATP production is up-regulated in order to satisfy the energetic requirements of further differentiation and development, and to support processes required for implantation [20]. Most of the information concerning the function of the mitochondrial organelle and its genome during this critical phase of development comes from experiments in animal models. To date, few studies using human embryos have been undertaken.

### The influence of female age on mtDNA quantity

Previous studies examining human mitochondria and mtDNA in relation to female reproductive aging have focused on the analysis of oocytes rather than embryos. Published results have not been entirely concordant, but most report that mtDNA levels either remain unchanged or decrease with advancing age [16, 21–22]. A reduction in the number of oocyte mitochondria with age has also been reported in older mice [23]. Other research has indicated that a decline in oocyte mtDNA copy number may be associated with ovarian pathology [1, 24]. During the current study a significant (P = 0.01) decline in mtDNA quantities was observed in cells from cleavage stage embryos generated by reproductively older women, compared to those from younger patients. Considering that the main wave of mtDNA replication is thought to start after blastocyst formation [3, 8], these observations at an early preimplantation stage are likely to be representative of the quantities of mtDNA that were present in the corresponding oocytes. Our data are therefore supportive of the notion that oocyte mtDNA levels decrease with advancing female age.

Interestingly, analysis of specimens from human blastocysts, just two days after the cleavage stage, revealed a trend in the opposite direction, with mtDNA levels increasing significantly with advancing female age. This association was apparent for both euploid and aneuploid blastocysts. It is likely that the elevated quantities of mtDNA observed are indicative of an increase in the number of mitochondria, although the relationship between the two factors is complicated by the fact that a single organelle may contain more than one copy of the mitochondrial genome.

It is well established that the likelihood of an oocyte producing a viable embryo is inversely correlated with the age of the mother. This is clearly demonstrated by the significant difference in the success rate of IVF treatment for older patients using their own oocytes, compared to patients utilising gametes donated by younger women. The increase in mtDNA with age seen at the blastocyst stage during the current study raises the question of whether mitochondria might play a direct role in the decline of female fertility with age. However, an answer to this important question is beyond the scope of the research reported here.

It is conceivable that elevated mtDNA levels are a consequence of a compensatory mechanism, aimed at normalisation of ATP generation in the face of growing numbers of compromised organelles of reduced function. Indeed, data obtained from animal models suggest a decline in the integrity of ‘older’ mitochondria and a consequent deterioration in the efficiency of ATP production [24, 25]. Mitochondria in the oocytes of older hamsters and mice have been shown to generate higher levels of reactive oxygen species (ROS), produce less ATP, and are therefore likely to have a reduced capacity to adequately support a dynamic process such as preimplantation development [26]. If a similar situation exists in humans, an increase in mitochondrial number may be necessary in the embryos of older women, in order for sufficient ATP levels to be maintained.

A decline in ATP synthetic capability with age could be related to an accumulation of mutations in the mitochondrial genome. An increase in the mtDNA content of human preimplantation embryos in response to mutation has previously been documented [27]. The location of the mtDNA in close proximity to ROS generated by the respiratory chain, coupled with a lack of histones and inferior DNA repair mechanisms, leaves the mitochondrial genome particularly vulnerable to mutation [8, 24]. In theory, the longer the oocyte remains in the ovary prior to fertilisation, the greater the opportunity for mtDNA mutation to occur. Several studies have shown a reduction in mitochondrial gene expression in oocytes that fail to fertilise after exposure to sperm and in embryos that undergo developmental arrest. An increase in the incidence of the common mitochondrial 4977 bp deletion, associated with ageing in various tissues, has also been noted in human oocytes [24, 28, 29]. However, in the current study, sequencing of the entire mitochondrial genome using NGS failed to detect an obvious increase in mutation load in embryos with high mtDNA levels. This finding argues against the possibility that mitochondrial mutation is driving replication of the organelle in embryos from older women.

It may be that high mtDNA levels are indeed indicative of compromised mitochondria, but that the underlying defects are unrelated to alterations in the DNA sequence. Alternatively, elevated quantities of mtDNA might be associated with increased metabolic requirements of the embryo, rather than organelles of suboptimal function. It is possible that embryos produced by older oocytes are under some form of stress and therefore have larger energy requirements. Functional experiments will be required to address these questions. Whatever the underlying basis, the current study has unequivocally demonstrated that female reproductive aging is associated with changes in the mtDNA content at the blastocyst stage.

### mtDNA and blastocyst chromosome status

Aneuploidy affects more than half of all human preimplantation embryos and is believed to be the most important cause of early embryonic demise [18]. The majority of chromosome abnormalities are derived from errors occurring during oogenesis (meiotic, female origin), but chromosome malsegregation is also common during the first few embryonic cell divisions following fertilisation (mitotic). Despite their frequency and clinical importance, the reasons for the high levels of meiotic and mitotic errors are still not fully understood.

As well as undergoing mtDNA quantification, all embryos analysed during this study had previously been tested for aneuploidy as part of routine PGD or PGS using a well-validated comprehensive chromosome screening method [30, 31]. A comparison of the cytogenetic (aCGH) and mitochondrial (real-time PCR) data produced demonstrated that, on average, biopsy specimens derived from aneuploid blastocysts contained significantly greater amounts of mtDNA than samples from embryos that were euploid (P = 0.025). These findings were confirmed using an alternative method (NGS) to assess an independent group of embryos. Importantly, the rise in mtDNA copy number seen in chromosomally abnormal embryos was additional to the association with female age, such that aneuploid blastocysts tended to have higher levels of mtDNA compared to chromosomally normal embryos derived from women of the same age.

It is plausible that variation in the quantity or functionality of mtDNA/mitochondria could have a direct effect on the accuracy of chromosome segregation. Mitochondrial metabolism factors, including ATP and the pyruvate dehydrogenase complex are essential for correct oocyte spindle assembly and chromosome alignment [32–34]. Furthermore, examination of oocytes from diabetic mice has demonstrated that damaged mitochondria are associated with aneuploidy. It is known that mitochondria are redistributed to spindles and microtubule organizing centres during cell division [35], presumably to ensure that the energy requirements of spindle formation and chromosome movement are satisfied. A link between mitochondrial distribution within the oocyte and chromosome congression on the meiotic spindle has been proposed [36]. Furthermore, it has been shown that embryos with high levels of chromosomal mosaicism, a consequence of errors occurring during the mitotic divisions following fertilisation, frequently contain mitochondria with low membrane potential [37].

It is unclear at this time whether aneuploidy in embryos with increased quantities of mtDNA is a direct consequence of deficiencies affecting the organelle, disrupting ATP production or other key functions, or whether altered mitochondrial number and aneuploidy are independent, downstream consequences of another issue, currently undefined, affecting the embryo or the oocyte. It is important to note that although the increased quantities of mtDNA associated with age and aneuploidy were only seen in blastocysts, the trigger for expansion may already exist in oocytes prior to fertilisation. Most of the aneuploidies observed in blastocysts are a consequence of errors occurring during female meiosis [30, 38], suggesting that factors that predispose to meiotic aneuploidy in oocytes might also have an effect on mtDNA replication during later embryonic stages.

### mtDNA and blastocyst implantation potential

In order to improve the efficiency of assisted reproductive treatments, superior methods for the identification of viable embryos are urgently required. The screening of embryos for cytogenetic abnormalities prior to transfer to the uterus allows the main cause of embryonic failure (i.e. aneuploidy) to be avoided. However, even the transfer of a morphologically ‘perfect’ embryo, which is additionally considered chromosomally normal following analysis of biopsied cells, cannot guarantee the initiation of a successful pregnancy (only about two thirds of such embryos actually produce a child). It is clear that additional elements play a role in embryo viability. Important factors might conceivably include mitochondrial number/capacity and accompanying effects on ATP content and/or metabolic activity [17]. As part of this investigation, the levels of mtDNA were retrospectively assessed in euploid cleavage and blastocyst stage embryos that had been transferred to the uterus following PGD or PGS and for which the clinical outcome was known.

The levels of mtDNA observed in biopsied cells were lower on average for blastocysts capable of establishing a clinical pregnancy compared to those that failed to implant after transfer (P = 0.007). This relationship was initially identified using quantitative PCR, but was subsequently verified using NGS. The association between mtDNA quantity and ability to produce a pregnancy was only clearly observed in embryo samples taken at the blastocyst stage of development. The increases in mtDNA content associated with loss of embryo viability were more dramatic than those related to age or aneuploidy.

Analysis of the mtDNA content data allowed the establishment of a threshold above which implantation of a chromosomally normal blastocyst was never observed. This cut-off remained valid regardless of other considerations such as embryo morphology or the clinic where the patients were receiving treatment. Approximately one-third of non-viable blastocysts had mtDNA levels above the threshold, suggesting that this factor represents an indicator of lethally compromised embryos, second only to aneuploidy in terms of prevalence and clinical importance. In order to confirm the predictive power of mtDNA measurement, an independent series of blastocysts were blindly assessed in a prospective manner. Once again, all euploid blastocysts with mtDNA levels above the threshold failed to implant (100%). Those with quantities of mtDNA in the normal range displayed a 59% implantation rate, which contrasts to 38% for the group as a whole.

The failure to detect a clear association between mtDNA levels and implantation potential for cleavage stage embryos suggests that the elevated quantities of mtDNA seen in a subset on non-viable blastocysts are a consequence of an expansion that occurs after day-3 post-fertilisation. An up-regulation in the expression of mtDNA replication factors is known to occur at the blastocyst stage, and this is generally considered to coincide with the first significant wave of mtDNA synthesis [3]. This may also be the time when excessive increases in mtDNA levels occur in some non-viable embryos.

Abnormally high levels of mtDNA at the blastocyst stage may be symptomatic of some form of stress that results in elevated energy requirements. This possibility would be consistent with the ‘quiet embryo hypothesis’, proposed by Leese, which suggests that viable embryos have relatively lower or ‘quiet’ metabolism, whereas those under stress, and of reduced developmental potential, tend to be more metabolically active [39].

It is of note that blastocysts with high mtDNA quantities, incapable of producing a viable pregnancy, were mostly generated by women aged 38 years or older. This observation is not surprising considering that an increase in mtDNA in relation to advancing female age had been observed. The association between female age and diminished embryo viability is well established and known to be primarily due to aneuploidy [40]. However, our findings suggest mitochondria represent an important additional factor.

### Conclusions

This study identified for the first time a clear association between mtDNA quantity and the ability of a human embryo to implant in the uterus. The discovery of a new biomarker of embryo viability, independent of standard assessments such as morphology, is a rare event and of great clinical potential. In the current study an mtDNA threshold was established, above which implantation failure was 100%. The data obtained suggests that embryo deficiencies associated with elevated mtDNA explain up to one-third of implantation failures affecting blastocysts diagnosed euploid. The defined mtDNA threshold does not appear to be altered by variation in the processes used by different fertility clinics, indicating that evaluation of mtDNA in embryos could form the basis of a simple, inexpensive and widely applicable clinical test.

Relationships between mtDNA content, female age and embryo chromosomal status were also demonstrated. The possibility that mtDNA content has a direct influence on embryo viability and the potential for a causal relationship with aneuploidy, and other factors related to reproductive senescence warrant further investigation.

## Materials and Methods

### Ethics statement

Ethical approval was obtained from Western IRB (20060680 and 20131473) and the NHS Health Research Authority (NRES Committee South Central). Two types of embryonic samples were assessed: blastomeres derived from cleavage stage embryos (3 days post-fertilisation of the oocyte) and TE cells from blastocysts (5–6 days post-fertilisation). All of the embryos included in this study underwent biopsy at the request of the couples who generated them, in order to analyse their chromosomes in the context of either preimplantation genetic screening (PGS) or preimplantation genetic diagnosis (PGD). The standard process used in our laboratory for this purpose involves whole genome amplification (WGA) followed by aCGH. The research described in this paper involved analysis of surplus amplified DNA only, leftover after PGD or PGS had been completed. The embryos were not subjected to any additional interventions and the clinical treatment of the patients was not altered as a result of this study. Informed patient consent for analysis of discarded amplified DNA was obtained under an approved protocol (see above).

### Patients and samples

Surplus WGA samples were derived from TE biopsies (typically consisting of 5–10 cells) from a total of 340 blastocysts. The blastocysts were produced by 161 couples of an average female age of 38 years (range 26–42 years). The excess WGA products from single blastomeres were obtained from a total of 39 cleavage stage embryos. These embryos were generated by 32 couples. The average female age of this patient group was 37.4 years (age range 29–42 years). IVF clinics located in the USA and the UK participated in this investigation.

### Embryo sampling and cytogenetic analysis preparation

Embryo micromanipulation, biopsy, and preparation of biopsied material for chromosome analysis were as described previously [18, 30]. All samples were analyzed with the use of a single, highly validated platform for microarray comparative genomic hybridisation (aCGH) [30, 31, 41–44].

### Microarray CGH

Chromosome analysis was carried out using 24Sure Cytochip V3 microarrays (Illumina Ltd., Cambridge, UK). The protocol used was as described in Fragouli *et al*., [18]. In brief, the procedure involved cell lysis and WGA (SurePlex, Rubicon Genomics, Ann Arbor, USA). This was followed by fluorescent labelling of amplified DNA samples, and two ‘reference’ DNAs (46,XY an 46,XX), and hybridization to the microarray. The microarrays were washed, scanned (InnoScan 710, Innopsys, Carbonne, France), and the resulting images analyzed using BlueFuse software (Illumina, Cambridge, UK). Using this approach, it was possible to determine the chromosome constitution of the blastomere or TE samples, allowing classification of the corresponding embryos as normal or aneuploid.

### Relative quantification of mtDNA copy number

mtDNA copy number quantification took place initially via fluorescent real-time PCR assessment of embryonic samples. These had previously undergone WGA (SurePlex, Rubicon, USA), as part of the cytogenetic analysis described above. A custom-designed TaqMan Assay (AATTTAACTGTTAGTCCAAAGAG, Life Technologies, UK) was used to target and amplify a specific mtDNA fragment (the mitochondrial 16s ribosomal RNA sequence [45]. Normalisation of input DNA took place with the use of an additional TaqMan Assay targeting the multicopy Alu sequence (YB8-ALU-S68) (AGCTACTCGGGAGGCTGAAGGCAGGA, Life Technologies, UK). The purpose of normalisation relative to a nuclear DNA sequence was to ensure that any variation in mtDNA levels related to technical issues (e.g. differences in the efficiency of WGA or the number of cells within the biopsy specimen) could be adjusted for. A multicopy sequence (i.e. Alu) was chosen for this purpose since, at the single cell level, single copy sequences may give spurious results due to factors such as allele drop-out (ADO). Each real-time PCR experiment included analysis of a reference DNA against which all samples were compared. The reference DNA was derived from a karyotypically normal male (46,XY) blastomere or TE sample, amplified via the SurePlex method (Rubicon, USA), and remained constant throughout the course of this study. A negative control (nuclease free H_2_O and PCR master-mix) was also included for both sets of amplifications. Triplicate amplification reactions were set up for both the mtDNA and Alu sequences. Each reaction contained 1 μl of whole genome amplified (SurePlex) embryonic DNA, 8 μl of nuclease-free H_2_O, 10 μl of TaqMan Universal Master-mix II (2X)/ no UNG (Life Technologies, UK) and 1 μl of the 20× TaqMan mtDNA or Alu assay (Life Technologies, UK), for a total volume of 20 μl. The thermal cycler used was a StepOne Real-Time PCR System (Life Technologies, UK), and the following conditions were employed: incubation at 50°C for 2 min, incubation at 95°C for 10 min and then 30 cycles of 95°C for 15 s and 60°C for 1min.

### Mitochondrial genome analysis via next generation sequencing

A group of 23 WGA products from euploid TE samples with varying levels of mtDNA (previously established via real-time PCR) underwent massively parallel DNA sequencing using a MiSeq and a HiSeq (Illumina, USA). The protocol was as suggested by the manufacturer (Illumina, USA). Library preparation involved the initial purification of SurePlex amplified products with the use of the Zymo DNA Clean & Concentrator (Zymo Research Corporation, Irvine, CA, USA), followed by quantification of DNA concentrations via the Qubit dsDNA HS Assay Kit (Life Technologies, USA). One nanogram of DNA was subsequently converted into dual-indexed sequencing libraries using the Nextera XT DNA Sample Preparation and Index Kits according to the manufacturer’s protocol (Illumina, USA).

The libraries were sequenced 2x150 cycles with dual indexing on an Illumina MiSeq using the MiSeq Reagent Kit v3 or 2x100 cycles with dual indexing on an Illumina HiSeq 2000 using the TruSeq PE Cluster Kit v3-cBot-HS and TruSeq SBS kit v3-HS for flow cell clustering and sequencing respectively (Illumina, USA).

Reads were aligned to the human genome hg19 using *bwa* [46] or *iSAAC* [47] for MiSeq and HiSeq sequencing runs respectively. After alignment, unmapped reads, duplicate reads, reads with low mapping scores and reads with greater than one mismatch with the reference genome were removed using BEDtools [48] and SAMtools [49]. The reference genome was divided into non-overlapping bins such that each bin contains 100 uniquely mapping 36mers across the genome [50] and the number of reads that mapped to each bin was counted. The bin read count was normalized based on GC content and an *in-silico* reference data set in order to remove bias. The copy number per bin was calculated according to the formula: $Copy\;Number=\frac{Bin\;Read\;Count}{Median\;Bin\;Read\;Count}\;X\;2$, where the median autosomal read count is expected to correspond to copy number two. A 13-bin sliding median was used to smooth bin-wise copy number values for each chromosome. Copy-number status for each chromosome was derived from the median of smoothed copy-number values across the chromosome.

After alignment, genomeCoverageBed files were generated using BEDTools and the fraction of total sequenced bases that aligned to the mitochondrial genome relative to the nuclear genome was calculated. Using SAMtools, the mitochondrial reads were extracted from the BAM files and analyzed with the online tool MitoBamAnnotator [51].

### mtDNA quantification via NGS

An additional 38 TE samples underwent NGS analysis in order for chromosome constitution assessment and mtDNA quantification to take place. A different type of NGS technology was used for the analysis of these 38 TE samples, involving the application of the PGM (Life Technologies, UK). For this purpose a different initial WGA approach involving the use of multiple displacement amplification (MDA) was employed. Briefly, all 38 TE samples were lysed by adding 2.5μl of alkaline lysis buffer (0.75 μl PCR-grade water [Promega, USA]; 1.25 μl DTT [0.1M] [Sigma, UK]; 0.5 μl NaOH [1.0M][Sigma, UK]), and then incubating them for 10 min at 65°C in a thermal-cycler. MDA whole genome amplification took place with the use of the Repli-g Midi Reaction kit (Qiagen, UK) as was suggested by the manufacturer. All samples were incubated in a thermal-cycler at 30°C for 120 minutes and 65°C for a further 5 minutes. The NGS procedure used is described in detail in Wells *et al*. [31].

### Statistical analysis

The relative amount of mtDNA in relation to the Alu sequence for both reference and test samples was determined by the equation 2-^Delta Delta Ct^. The Delta Ct for reference and test samples was the end result of a data normalisation process. This involved the calculation of the Delta Ct for reference and test loci (Ct-mtDNA minus Ct-Alu), and the adjustment of the test samples values in relation to the reference DNA sample (Delta Ct plus Normalisation factor) [52]. Statistical analysis of the final mtDNA values utilised unpaired two-tailed t-tests. Parameters which were compared during this study included female age (younger vs. older), embryo chromosome status (normal vs. aneuploid), and embryo viability/implantation potential (ongoing pregnancy vs. failure to implant).

Relative quantification of mtDNA using NGS involved determination of the number of DNA sequence reads attributable to the mitochondrial genome as a fraction of the total number of reads. The great majority of DNA fragments sequenced are derived from the nuclear genome and provide a control for the number of cells in the biopsy specimen.

## References

[1] May-Panloup P, Chrétien MF, Jacques C, Vasseur C, Malthièry Y, et al (2005) Low oocyte mitochondrial DNA content in ovarian insufficiency. Hum Reprod 20: 593–597. 15608038 10.1093/humrep/deh667

[2] Dumollard R, Carroll J, Duchen MR, Campbell K, Swann K (2009) Mitochondrial function and redox state in mammalian embryos. Semin Cell Dev Biol 20: 346–353. 19530278 10.1016/j.semcdb.2008.12.013

[3] St John JC, Facucho-Oliveira J, Jiang Y, Kelly R, Salah R (2010) Mitochondrial DNA transmission, replication and inheritance: a journey from the gamete through the embryo and into offspring and embryonic stem cells. Hum Reprod Update 16: 488–509. 10.1093/humupd/dmq002 20231166

[4] Bentov Y, Casper RF (2013) The aging oocyte—can mitochondrial function be improved? Fertil Steril 99: 18–22. 10.1016/j.fertnstert.2012.11.031 23273985

[5] Tilly JL, Sinclair DA (2013) Germline energetics, aging, and female infertility. Cell Metab 17: 838–850. 10.1016/j.cmet.2013.05.007 23747243 PMC3756096

[6] Anderson S, Bankier AT, Barrell BG, de Bruijn MH, Coulson AR, et al (1981) Sequence and organization of the human mitochondrial genome. Nature 290: 457–465. 7219534 10.1038/290457a0

[7] Palmer CS, Osellame LD, Stojanovski D, Ryan MT (2011) The regulation of mitochondrial morphology: intricate mechanisms and dynamic machinery. Cell Signal 23: 1534–1545. 10.1016/j.cellsig.2011.05.021 21683788

[8] Eichenlaub-Ritter U, Wieczorek M, Lüke S, Seidel T (2011) Age related changes in mitochondrial function and new approaches to study redox regulation in mammalian oocytes in response to age or maturation conditions. Mitochondrion 11: 783–796. 10.1016/j.mito.2010.08.011 20817047

[9] Chen X, Prosser R, Simonetti S, Sadlock J, Jagiello G, et al (1995) Rearranged mitochondrial genomes are present in human oocytes. Am J Hum Genet 57: 239–247. 7668249 PMC1801549

[10] Motta PM, Nottola SA, Makabe S, Heyn R (2000). Mitochondrial morphology in human fetal and adult germ cells. Hum Reprod 15: 129–147. 11041520 10.1093/humrep/15.suppl_2.129

[11] Cummins JM (2000) Fertilization and elimination of the paternal mitochondrial genome. Hum. Reprod 15: 92–101. 11041517 10.1093/humrep/15.suppl_2.92

[12] Steuerwald N, Barritt JA, Adler R, Malter H, Schimmel T, et al (2000) Quantification of mtDNA in single oocytes, polar bodies and subcellular components by real-time rapid cycle fluorescence monitored PCR. Zygote 8: 209–215. 11014500 10.1017/s0967199400001003

[13] Reynier P, May-Panloup P, Chrétien MF, Morgan CJ, Jean M, et al (2001) Mitochondrial DNA content affects the fertilizability of human oocytes. Mol Hum Reprod 7: 425–429. 11331664 10.1093/molehr/7.5.425

[14] Barritt JA, Kokot M, Cohen J, Steuerwald N, Brenner CA (2002) Quantification of human ooplasmic mitochondria. Reprod Biomed Online 4: 243–247. 12709274 10.1016/s1472-6483(10)61813-5

[15] Lin DP, Huang CC, Wu HM, Cheng TC, Chen CI, et al (2004) Comparison of mitochondrial DNA contents in human embryos with good or poor morphology at the 8-cell stage. Fertil Steril 81: 73–79. 14711547 10.1016/j.fertnstert.2003.05.005

[16] Chan CC, Liu VW, Lau EY, Yeung WS, Ng EH, et al (2005) Mitochondrial DNA content and 4977 bp deletion in unfertilized oocytes. Mol Hum Reprod 11: 843–846. 16421213 10.1093/molehr/gah243

[17] Van Blerkom J, Davis PW, Lee J (1995) ATP content of human oocytes and developmental potential and outcome after in-vitro fertilization and embryo transfer. Hum Reprod 10: 415–424. 7769073 10.1093/oxfordjournals.humrep.a135954

[18] Fragouli E, Alfarawati S, Spath K, Jaroudi S, Sarasa J, et al (2013). The origin and impact of embryonic aneuploidy. Hum Genet 132: 1001–1013. 10.1007/s00439-013-1309-0 23620267

[19] Van Blerkom J (2011) Mitochondrial function in the human oocyte and embryo and their role in developmental competence. Mitochondrion 11: 797–813. 10.1016/j.mito.2010.09.012 20933103

[20] Houghton F (2006) Energy metabolism of the inner cell mass and trophectoderm of the mouse blastocyst. Differentiation 74: 11–18. 16466396 10.1111/j.1432-0436.2006.00052.x

[21] Barritt JA, Cohen J, Brenner CA (2000) Mitochondrial DNA point mutation in human oocytes is associated with maternal age. Reprod Biomed Online 1: 96–100. 12804188 10.1016/s1472-6483(10)61946-3

[22] Konstantinidis M, Alfarawati S, Hurd D, Paolucci M, Shovelton J, et al (2014) Simultaneous assessment of aneuploidy, polymorphisms, and mitochondrial DNA content in human polar bodies and embryos with the use of a novel microarray platform. Fertil Steril: in press. 10.1016/j.fertnstert.2014.07.1233 25217868

[23] Narita A (1995) Endogenous factors affecting sterility in oocytes of aged animals. Jap J Fertil Steril 40: 57–65.

[24] Duran HE, Simsek-Duran F, Oehninger SC, Jones HW Jr, Castora (2011) The association of reproductive senescence with mitochondrial quantity, function, and DNA integrity in human oocytes at different stages of maturation. Fertil Steril 96: 384–388. 10.1016/j.fertnstert.2011.05.056 21683351

[25] Piko L, Taylor KD (1987) Amounts of mitochondrial DNA and abundance of some mitochondrial gene transcripts in early mouse embryos. Dev Biol 123: 364–374. 2443405 10.1016/0012-1606(87)90395-2

[26] Simsek-Duran F, Li F, Ford W, Swanson RJ, Jones HW Jr, et al (2009) The effect of aging on the metabolic function and structure of mitochondria in hamster oocytes. FASEB J 855: 10.

[27] Monnot S, Samuels DC, Hesters L, Frydman N, Gigarel N, et al (2013) Mutation dependance of the mitochondrial DNA copy number in the first stages of human embryogenesis. Hum Mol Genet 22: 1867–1872. 10.1093/hmg/ddt040 23390135

[28] Hsieh RH, Au HK, Yeh TS, Chang SJ, Cheng YF, et al (2004) Decreased expression of mitochondrial genes in human unfertilized oocytes and arrested embryos. Fertil Steril 81: 912–918. 15019829 10.1016/j.fertnstert.2003.11.013

[29] Hsieh RH, Tsai NM, Au HK, Chang SJ, Wei YH, et al (2002) Multiple rearrangements of mitochondrial DNA in unfertilized human oocytes. Fertil Steril 77: 1012–1017. 12009360 10.1016/s0015-0282(02)02994-1

[30] Fragouli E, Alfarawati S, Daphnis DD, Goodall NN, Mania A, et al (2011) Cytogenetic analysis of human blastocysts with the use of FISH, CGH and aCGH: scientific data and technical evaluation. Hum Reprod 26:480–490 10.1093/humrep/deq344 21147821

[31] Wells D, Kaur K, Grifo J, Glassner M, Taylor JC, et al (2014) Clinical utilisation of a rapid low-pass whole genome sequencing technique for the diagnosis of aneuploidy in human embryos prior to implantation. J Med Genet 51: 553–562. 10.1136/jmedgenet-2014-102497 25031024 PMC4112454

[32] Zhang X, Wu XQ, Lu S, Guo YL, Ma X (2006) Deficit of mitochondria- derived ATP during oxidative stress impairs mouse MII oocyte spindles. Cell Res 16: 841–850. 16983401 10.1038/sj.cr.7310095

[33] Choi WJ, Banerjee J, Falcone T, Bena J, Agarwal A, et al (2007) Oxidative stress and tumor necrosis factor-induced alterations in metaphase II mouse oocyte spindle structure. Fertil Steril 88:1220–1231. 17601599 10.1016/j.fertnstert.2007.02.067

[34] Johnson MT, Freeman EA, Gardner DK, Hunt PA (2007) Oxidative metabolism of pyruvate is required for meiotic maturation of murine oocytes in vivo. Biol Reprod 77: 2–8. 17314311 10.1095/biolreprod.106.059899

[35] Yu Y, Dumollard R, Rossbach A, Lai FA, Swann K (2010) Redistribution of mitochondria leads to bursts of ATP production during spontaneous mouse oocyte maturation. Journal of Cellular Physiology 224: 672–680. 10.1002/jcp.22171 20578238 PMC3149123

[36] Wang Q, Ratchford AM, Chi MM, Schoeller E, Frolova A, et al (2009) Maternal diabetes causes mitochondrial dysfunction and meiotic defects in murine oocytes. Mol Endocrinol 23: 1603–1612. 10.1210/me.2009-0033 19574447 PMC2754898

[37] Wilding M, De Placido G, DeMatteo L, Marino M, Alviggi C, et al (2003) Chaotic mosaicism in human preimplantation embryos is correlated with a low mitochondrial membrane potential, Fertil Steril 79: 340–346. 12568843 10.1016/s0015-0282(02)04678-2

[38] Capalbo A, Wright G, Elliott T, Ubaldi FM, Rienzi L et al (2013) FISH reanalysis of inner cell mass and trophectoderm samples of previously array-CGH screened blastocysts shows high accuracy of diagnosis and no major diagnostic impact of mosaicism at the blastocyst stage. Hum Reprod 28: 2298–2307. 10.1093/humrep/det245 23739221

[39] Leese HJ (2002) Quiet please, do not disturb: a hypothesis of embryo metabolism and viability. Bioessays 24: 845–849. 12210521 10.1002/bies.10137

[40] Harton GL, Munné S, Surrey M, Grifo J, Kaplan B, McCulloh DH, et al (2013) Diminished effect of maternal age on implantation after preimplantation genetic diagnosis with array comparative genomic hybridization. Fertil Steril 100: 1695–1670 10.1016/j.fertnstert.2013.07.2002 24034939

[41] Magli MC, Montag M, Koster M, Muzi L, Geraedts J, et al (2011) Polar body array CGH for prediction of the status of the corresponding oocyte. Part II: technical aspects. Hum Reprod 26:3181–3185. 10.1093/humrep/der295 21908464 PMC3196879

[42] Gutierrez-Mateo C, Colls P, Sanchez-Garcıa J, Escudero T, Prates R, et al (2011) Validation of microarray comparative genomic hybridization for comprehensive chromosome analysis of embryos. Fertil Steril 95:953–958. 10.1016/j.fertnstert.2010.09.010 20971462

[43] Christopikou D, Tsorva E, Economou K, Shelley P, Davies S, et al (2013) Polar body analysis by array comparative genomic hybridization accurately predicts aneuploidies of maternal meiotic origin in cleavage stage embryos of women of advanced maternal age. Hum Reprod 28: 1426–1434. 10.1093/humrep/det053 23477909

[44] Mertzanidou A, Spits C, Nguyen HT, Van de Velde H, Sermon K (2013) Evolution of aneuploidy up to Day 4 of human preimplantation development. Hum Reprod 28: 1716–1724. 10.1093/humrep/det079 23526301

[45] Fregel R, Almeida M, Betancor E, Suarez NM, Pestano J (2011) Reliable nuclear and mitochondrial DNA quantification for low copy number and degraded forensic samples. Forensic Science International: Genetics Supplement Series 3: e303–e304.

[46] Li H, Durbin R (2009) Fast and accurate short read alignment with Burrows—Wheeler transform. Bioinformatics 25: 1754–1760. 10.1093/bioinformatics/btp324 19451168 PMC2705234

[47] Raczy C, Petrovski R, Saunders CT, Chorny I, Kruglyak S et al (2013) Isaac: ultra-fast whole-genome secondary analysis on Illumina sequencing platforms. Bioinformatics 29:2041–2043 10.1093/bioinformatics/btt314 23736529

[48] Quinlan AR, Hall IM (2010) BEDTools: A flexible suite of utilities for comparing genomic features, Bioinformatics 26: 841–842. 10.1093/bioinformatics/btq033 20110278 PMC2832824

[49] Li H, Handsaker B, Wysoker A, Fennell T, Ruan J, et al (2009) The Sequence alignment/map (SAM) format and SAMtools. Bioinformatics 25: 2078–2079. 10.1093/bioinformatics/btp352 19505943 PMC2723002

[50] Baslan T, Kendall J, Rodgers L, Cox H, Riggs M, et al (2012) Genome-wide copy number analysis of single cells. Nat Protoc 7: 1024–1041. 10.1038/nprot.2012.039 22555242 PMC5069701

[51] Zhidkov I, Nagar T, Mishmar D, Rubin E (2011) MitoBamAnnotator: A web-based tool for detecting and annotating heteroplasmy in human mitochondrial DNA sequences. Mitochondrion 11: 924–928. 10.1016/j.mito.2011.08.005 21875693

[52] Schmittgen TD, Livak KJ (2008) Analyzing real-time PCR data by the comparative CT method. Nat Protoc 3: 1101–1108. 18546601 10.1038/nprot.2008.73

